# Advancing cellulose degradation through synthetic biology: engineered pathways and microbial systems for sustainable biomass conversion

**DOI:** 10.1186/s40104-025-01328-0

**Published:** 2026-01-20

**Authors:** Xingqi Liu, Jianping Quan, Ying Li, Xiaofan Wang, Jiangchao Zhao

**Affiliations:** 1 College of Animal Science, South China Agricultural University/Guangdong Laboratory for Lingnan Modern Agriculture, Animal Functional Microbiome Lab/State Key Laboratory of Swine and Poultry Breeding Industry/National Engineering Research Center for Breeding Swine Industry/Guangdong Provincial Key Laboratory of Animal Nutrition Control, Guangzhou, Guangdong 510642 China; 2 Guangdong Provincial Key Laboratory of Animal Molecular Design and Precise Breeding, School of Animal Science and Technology, Foshan University, Foshan, 528225 China

**Keywords:** Cellulose degradation, Cellulosome, CRISPR-Cas9, Gene editing, Synthetic biology

## Abstract

Fiber, the most abundant organic polymer in nature, is widely recognized as a foundational sustainable material with diverse applications across industrial, medical, and consumer domains. Owing to its renewability and widespread availability, it also serves as a critical alternative energy source in agriculture, enabling more sustainable livestock production through the efficient conversion of fibrous feedstuffs, thereby supporting the principles of a circular bioeconomy. Cellulose, which constitutes up to 80% of plant fiber, contains tightly packed crystalline regions that confer strong resistance to microbial degradation. Other key obstacles to efficient cellulose digestion in the gut include the absence of critical cellulolytic genes, low enzymatic activity, a lack of natural activators, and the presence of cellulase inhibitors. Synthetic biology provides innovative molecular-level strategies to overcome key technical barriers in cellulose degradation. These approaches employ targeted modifications at nucleic acid and protein levels, including the introduction of engineered genes, synthetic regulators, and optimized enzymes, to develop high-performance microbial systems with enhanced cellulose-degrading capabilities. Furthermore, genetic modifications like the knockout of inhibitory genes and knock-in of activator genes, combined with rational redesign of multi-enzyme complexes, can significantly improve the secretion and catalytic efficiency of cellulases. When integrated with artificial intelligence, synthetic biology enables predictive screening and precision engineering of microbial strains for highly efficient cellulose degradation. This review comprehensively summarizes recent advances in synthetic biology approaches for improving cellulose degradation and highlights how these tools can optimize fiber utilization in sustainable agricultural and industrial applications.

## Introduction

Fiber, the most abundant organic polymer in nature, serves as a crucial framework material of the biosphere [1]. It is ubiquitously found in a wide variety of plant materials, including alfalfa hay, straw, corn husks, as well as in agricultural processing byproducts like wheat bran and bagasse [2–5]. In 2024, the production of straw biomass exceeded 5 billion tons, an increase of approximately 24% compared to 2020, making it one of the most promising materials in the future [6]. Fiber serves as a critical material with extensive applications across multiple fields and daily life, including concrete reinforcement in industrial engineering, production of everyday necessities, medical devices, and tissue engineering scaffolds in biomedicine, as well as composite materials in aerospace [7–10].

In animal production, fiber is utilized by ruminants, monogastric animals, and poultry [11–13], serving as a significant energy source that can fulfill up to 70% of the energy requirements for ruminants and typically constitutes between 30% and 40% or more of their total dry matter intake [14–16]. For monogastric animals, especially pigs, which are the largest in body size, fiber, even if not a primary nutrient source, contributes 5%–28% of energy [17]. Currently, fiber accounts for approximately 10% of the diet in adult monogastric animals, but it has the potential to increase to 18% in the future to replace the energy contribution from grains such as corn [18]. In the context of increasingly frequent extreme weather events and natural disasters, addressing food shortages has become increasingly urgent. In this review, we will briefly describe the characteristics of fiber degradation in economically significant livestock species, especially in monogastric animals, the current factors limiting the large-scale use of fibrous feed, and focus on how synthetic biology can facilitate cellulose degradation to expand its application in animal husbandry, develop sustainable green microbial products, and promote the circular bioeconomy.

The degradation processes and efficiency of fiber vary significantly across animal species. Ruminants utilize microorganisms in the rumen to degrade fiber into volatile fatty acids (VFAs) that can be utilized by the host, along with byproducts such as carbon dioxide and methane [14]. Monogastric animals also rely on fiber-degrading microbial communities located in the gastrointestinal tract to ferment fiber into short-chain fatty acids (SCFAs), a process that primarily occurs in the caecum and colon [12]. Although fiber provides limited direct energy to monogastric animals, its unique and complex carbohydrate structures play an essential regulatory role in host physiology by actively shaping beneficial microbial ecosystems, maintaining gut health, and modulating critical communication pathways such as the gut-brain axis [19–21].

Fermentation of fiber in the caecum can regulate intestinal pH, thereby promoting Ca^2+^ uptake, activating the Wnt signaling pathway to alter the number of osteoblasts and indirectly regulate bone metabolism [13], thus maintaining skeletal health in livestock and poultry. Intake of a 5% high-fiber diet can alleviate jejunal hot stress caused by low-fiber feeding in growing-finishing pigs, reduce the need for antioxidant substances, and improve production performance [22]. The short-chain fatty acids produced by fermentation can stimulate G protein-coupled receptors (GPCRs), inhibit histone deacetylases (causing changes in gene transcription), and affect mucosal immune regulation, increasing the functional number of T cells and reducing the production of inflammatory factors [23]. The fiber metabolites such as butyrate can promote the proliferation of goblet cells in the pig caecum and increase the length of the caecum [24]. Therefore, beyond its role in energy provision, enhancing the efficiency of fiber degradation, from the perspectives of health and animal welfare, supports sustainable growth and overall productivity in livestock.

### Distribution, structure, and biological degradation of cellulose

The efficient fiber degradation is of critical importance and represents an important research objective in practical livestock production. However, achieving high degradation efficiency remains challenging due to the inherent composition and intricate structure. Its main components, cellulose, hemicellulose, and lignin, vary significantly across plant parts. Typically, cellulose comprises 40%–80% of fiber, hemicellulose 15%–25%, and lignin 5%–15% [3]. These three components assemble into a highly crystalline complex termed lignocellulose [25], which is highly recalcitrant to degradation and necessitates synergistic action by diverse microbial communities within the gut.

**Fig. 1:**
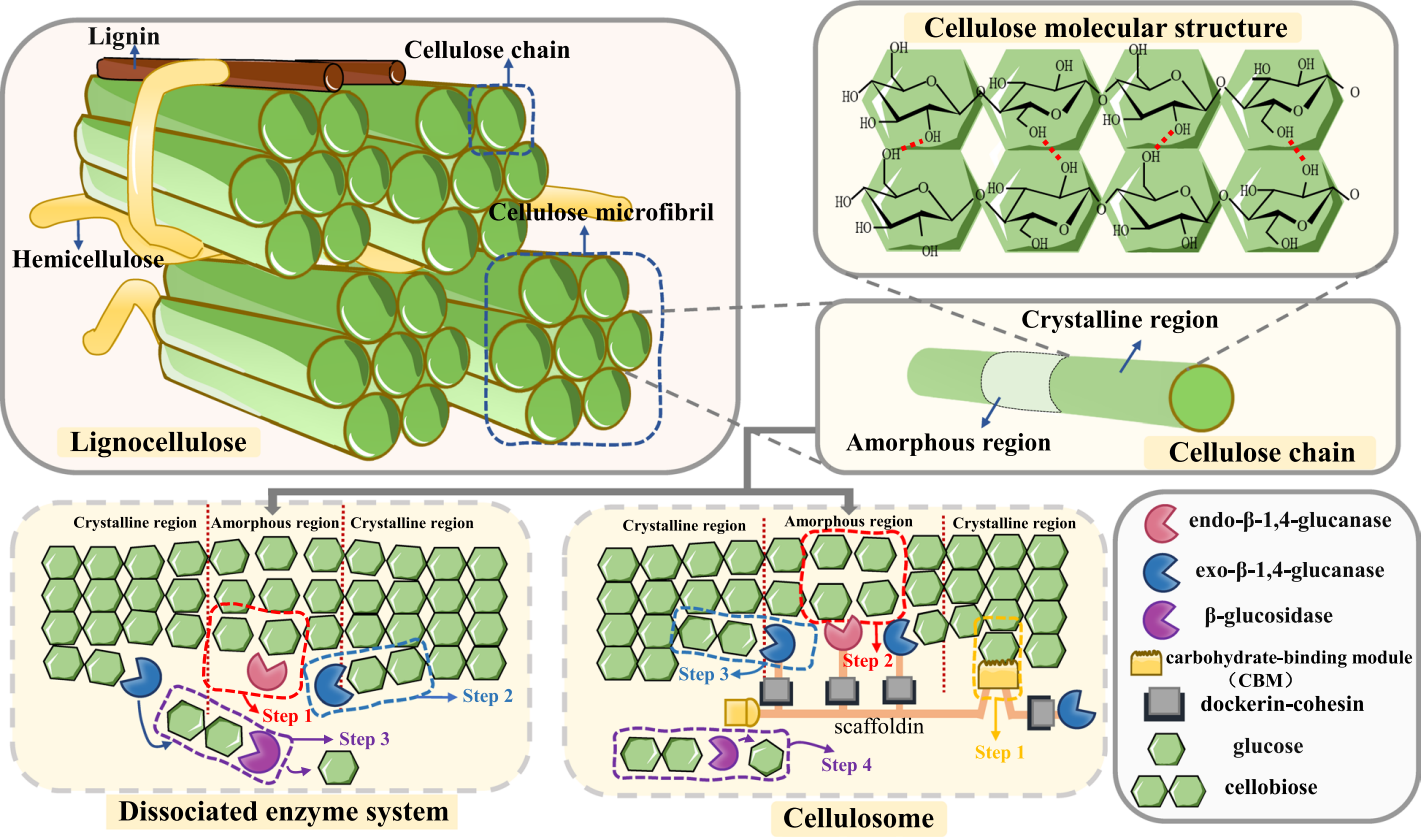
Mechanisms of cellulose degradation. Cellulose is a linear polymer that assembles into microfibrils, which further associate with hemicellulose and lignin to form recalcitrant lignocellulose. Along the cellulose chain, amorphous and crystalline regions alternate periodically. The crystalline regions, stabilized by extensive intra- and intermolecular hydrogen bonding, exhibit particularly high resistance to degradation. Cellulose degradation occurs primarily through two main pathways. The first involves the synergistic action of free enzymes: endoglucanases (EG), cellobiohydrolases (CBH), and β-glucosidases (BG) collaboratively hydrolyze cellulose extracellularly. The second mechanism employs cellulosomes, multi-enzyme complexes where a scaffoldin protein bears catalytic modules. Carbohydrate-binding module (CBM) mediates cellulose recognition, followed by degradation via bound EG and CBH, yielding cellobiose, which is subsequently cleaved to glucose by free BG. Abbreviations: EG, Endo-β-1,4-glucanase; CBH, Exo-β-1,4-glucanase; BG, β-glucosidase; R, reducing end; NR, non-reducing end; CBM, carbohydrate-binding module

Although cellulose plays an important role in these materials due to its predominant content, environmental friendliness, and biodegradability, it exhibits inherent resistance to degradation [26–28]. It comprises numerous D-pyranose glucose units linked together by β-1,4-glycosidic bonds to form chains of cellulose [29] (Fig. 1). These chains are further organized into dense microfibrils through hydrogen bonding between hydroxyl groups on the D-glucose residues, along with van der Waals forces and electrostatic interactions [30]. Due to variations in polymerization degree, cellulose is structurally categorized into amorphous and crystalline regions [31]. In the amorphous regions, cellulose molecules are held together by weak intermolecular forces and exhibit considerable spacing. This loose and disordered structural arrangement enhances their accessibility and susceptibility to degradation. Conversely, the crystalline region is characterized by strong intramolecular hydrogen bonds as well as robust intermolecular hydrogen bonds and van der Waals forces. This leads to significant binding strength, reduced intermolecular spacing, high molecular orientation and a tightly ordered structure [29–31]. Such structural integrity confers resistance against degradation and is regarded as one of the principal factors limiting the efficiency of cellulose breakdown.

Efficient cellulose degradation is critical for the utilization of fibrous resources. In livestock and poultry, cellulose breakdown is dependent on cellulolytic microorganisms [32]. Although physical and chemical methods are also employed for in vitro fiber treatment, their efficacy is constrained by inter-individual variability, as well as high energy expenditure and environmental concerns [33]. Livestock rely on a diverse community of cellulose-degrading microorganisms including both fungi and bacteria to break down fibrous plant material [34]. The microbial degradation of cellulose is fundamentally an enzymatic process that involves the synergistic action of multiple enzymes. For instance, lytic polysaccharide monooxygenases (LPMOs) can collaborate with endoglucanases to facilitate the breakdown of cellulose [35]. Microorganisms recognize and adsorb cellulose through specific binding domains located on their cell surfaces or via particular structural functions. Subsequently, they produce various enzymes to disrupt the structure of cellulose [11, 36–38]. Different types of cellulose-degrading microorganisms employ distinct mechanisms for degradation and yield varying metabolites [11, 14, 39–69] (Table 1).

**Table 1:** Degradation mechanisms and degradation products of different cellulose degrading bacteria

Variety		Species	Mechanism		Products	Reference
Bacteria (Aerobic)	*Bacillus*	*Bacillus subtilis* *Bacillus licheniformis* *Bacillus cereus*	Dissociated enzyme system	endo-β-1,4-glucanase exo-β-1,4-glucanase β-glucosidase	CO_2_, H_2_O, ATP	[39–42]
	*Sorangium*	*Sorangium arenae* *Sorangium ambruticinum* *Sorangium bulgaricum*			CO_2_, H_2_O, ATP	[43]
	*Myxococcus*	*Myxococcus cruentus* *Myxococcus stipitatus* *Myxococcus fulvus*	Outer membrane vesicle (OMV)	endo-β-1,4-glucanase exo-β-1,4-glucanase β-glucosidase	CO_2_, H_2_O, ATP	[44–46]
	*Cytophaga*	*Cytophaga hutchinsonii*				[47, 48]
Bacteria (Anaerobic)	*Fibrobacter*	*Fibrobacter succinogenes*			Succinate, CO_2_, H_2_	[49]
	*Clostridium*	*Clostridium thermocellum* *Clostridium cellulolyticum* *Clostridium cellulovorans*	Cellulosomes: non catalytic subunit catalytic subunit	endo-β-1,4-glucanase exo-β-1,4-glucanase β-glucosidase (free) primary scaffoldin adaptor scaffoldin anchoring scaffoldin	CO_2_, H_2_, SCFAs	[50–52]
	*Ruminococcus*	*Ruminococcus flavefaciens* *Ruminococcus champanellensis* *Ruminococcus albus*			CO_2_, H_2_, VFAs	[53–55]
Bacteria (Actinomycetes)	*Streptomyces*	*Streptomyces laurentii* *Streptomyces clavifer* *Streptomyces lividans*	Dissociated enzyme system	endo-β-1,4-glucanase exo-β-1,4-glucanase β-glucosidase	CO_2_, H_2_O, ATP	[56, 57]
	*Thermobifida*	*Thermobifida fusca* *Thermobifida alba* *Thermobifida halotolerans*			CO_2_, H_2_O, ATP	[58–60]
Fungi (Anaerobic)	*Neocallimastix*	*Neocallimastigomycota*	Cellulosomes: non catalytic subunit catalytic subunit	endo-β-1,4-glucanase exo-β-1,4-glucanase β-glucosidase	CO_2_, H_2_, SCFAs	[61, 62]
Fungi (Aerobic)	*Trichoderma*	*Trichoderma reesei*	Dissociated enzyme system	endo-β-1,4-glucanase exo-β-1,4-glucanase β-glucosidase	CO_2_, H_2_O, ATP	[63]
	*Aspergillus*	*Aspergillus niger* *Aspergillus oryzae*				
	*Penicillium*	*Penicillium simplicissimum* *Penicillium chrysogenum*				[64, 65]
	*Myceliophthora*	*Myceliophthora thermophila*		endo-β-1,4-glucanase exo-β-1,4-glucanase β-glucosidase	CO_2_, H_2_O, ATP	[66–69]
	*Thermomyces*	*Thermomyces lanuginosus* *Irpex lacteus*				
	*Phanerochaete*	*Polyporus ciliatus*			CO_2_, H_2_O, ATP	

*SCFAs* Short-chain fatty acids, *VFAs* Volatile fatty acids, *ATP* Adenosine triphosphate

Aerobic bacteria and fungi typically employ dissociated enzyme systems to degrade cellulose [11, 70, 71]. This enzymatic degradation is a synergistic process facilitated by three primary enzyme classes [72, 73]. Endo-β-1,4-glucanase (EG) initiates hydrolysis by randomly cleaving internal β-1,4-glycosidic bonds within amorphous regions, generating new chain ends and creating access points for subsequent enzymes [74]. Exo-β-1,4-glucanase (CBH) then attacks these exposed ends, predominantly releasing cellobiose units from both amorphous and crystalline regions, with notable efficacy against recalcitrant crystalline cellulose. It exhibits high efficacy against crystalline cellulose and is considered the rate-limiting enzyme in the degradation process [75]. Finally, β-glucosidase (BG) completes saccharification by hydrolyzing cellobiose and short-chain oligomers into glucose monomers [76], thereby preventing product inhibition of EG and CBH and ensuring efficient substrate conversion [77, 78] (Fig. 1).

The degradation of cellulose by aerobic bacteria relies on extracellular cellulase systems, and the enzyme activity of these systems varies among different strains. Compared with aerobic bacteria, fungi possess more efficient and comprehensive cellulase systems. However, the expression of cellulase genes is tightly regulated, including carbon source induction and carbon catabolite repression (CCR), transcriptional regulation and epigenetic regulation. Their regulatory mechanisms have not been fully elucidated. In microorganisms, there exist inhibitors of cellulase activity, including *CRE1*, *ACE1*, and there are also cases of deletion of cellulase activators such as *XYR1*, *ACE2*, and *ACE3* [79–83]. Some microorganisms also lack genes encoding exo-glucanase, and the expression of endo-glucanase genes is incomplete [84], which severely limits the efficiency of cellulose degradation.

Anaerobic bacteria and certain anaerobic fungi, such as the rumen fungus *Caecomyces churrovis*, degrade cellulose with the assistance of cellulosomes, which are multi-enzyme complexes [85–87] (Fig. 1). Cellulosomes consist of two main components: a catalytic subunit and a non-catalytic subunit. The non-catalytic subunit, also known as scaffoldin, serves as a structural scaffold within the cellulosome. Scaffoldin is composed of multiple cohesin domains, whose mechanical stability can influence the overall activity of the cellulosome [88]. The catalytic subunit is assembled through carbohydrate-binding modules (CBMs), dockerins, and various enzymes primarily glycoside hydrolases [89]. This catalytic subunit is firmly anchored to the scaffoldin protein via interactions between dockerins and cohesins [90]. Upon completion of assembly, the cellulosome enhances cellulose degradation through a multilayered synergistic mechanism [91]. The scaffoldin attaches to the cell surface via Dockerin II while cellulose recognition occurs through CBM. Subsequently, cellulose is degraded by the catalytic subunits (cellulases) on the brace protein, following a degradation sequence similar to that of the free enzyme system. EG on the scaffoldin first randomly cleaves the internal β-1,4-glycosidic bonds of the cellulose chain, breaking it into oligosaccharides of varying lengths. Then, CBH sequentially cleaves cellobiose units from the non-reducing ends of the chains, depolymerizing the long-chain cellulose. Finally, the free BG is responsible for hydrolyzing cellobiose into glucose monomers [88–91]. Anaerobic bacteria are the main force responsible for degrading cellulose in the animal gut [92, 93]. Nevertheless, the degradation efficiency exhibited by these microorganisms is markedly lower than that observed in their aerobic counterparts. During degradation, these bacteria secrete cellulosomes, which can physically disrupt the structure of lignocellulose. This increases the contact area between enzymes and substrates, enhancing degradation efficiency. Cellulosomes possesses multiple CBMs that exhibit strong affinity for cellulose upon recognition, firmly anchoring it to the microfiber surface [94]. Concentrated attacks on cellulose regions around anchor points sever numerous cellulose chains, disrupting the hydrogen bond network between fiber bundles and loosening the structure. Due to the high molecular weight of the cellulase complexes themselves, their sustained anchoring may also exert mechanical stress [95]. This process results in the separation of individual cellulose microfibrils from the primary fiber bundle. Consequently, the fiber bundle becomes irregular, branched, and disorganized, resembling the frayed end of a torn rope. This transformation enhances accessibility for free enzymes to degrade terminal fibrin disaccharides.

Notably, *Cytophaga hutchinsonii* and *Fibrobacter succinogenes* exhibit distinct degradation strategies [36, 96, 97] (Table 1). Studies demonstrated that *Fibrobacter succinogenes* degrades cellulose by utilizing outer membrane vesicles (OMVs) enriched in carbohydrate-active enzymes (CAZymes). Outer membrane proteins, periplasmic proteins, and cellulose-related protein complexes have been identified within these vesicles [96–98]. These components enable *Fibrobacter succinogenes* to target amorphous regions of cellulose; however, the mechanisms by which outer membrane proteins affect crystalline cellulose remain to be elucidated [99]. *Cytophaga hutchinsonii* degrades cellulose through the action of outer membrane proteins. Unlike *Fibrobacter succinogenes*, this organism exhibits motility along the cellulose surface and is capable of cleaving its structurally distinct regions. Degradation is primarily facilitated by outer membrane proteins that peel single chains of cellulose from crystalline areas and transport them to the periplasmic space, where cellulases further degrade these chains [99, 100].

### Synthetic biology offers innovative strategies to enhance cellulose degradation

Given the bottlenecks described above in microbial cellulose degradation, including low cellulase activity, the absence of activating elements, and the presence of inhibitory factors, synthetic biology currently offers a highly promising approach to address these challenges. This approach employs targeted modifications at the nucleic acid and protein levels, through the introduction of engineered genes, synthetic regulators, and optimized enzymes, to construct efficient microbial systems capable of enhanced cellulose breakdown, including designed enzyme complexes and specialized chassis strains [101, 102]. For example, engineered strains such as *Bacillus subtilis* demonstrate significantly accelerated cellulose degradation rates, modified *Lactobacillus* strains exhibit improved resilience to extreme temperatures, pH variations, and elevated salinity, achieving peak enzyme activity under specific conditions, optimized variant *Corynebacterium glutamicum* enables higher target product yields with minimized synthesis of metabolic byproducts [103]. To date, significant breakthroughs have been achieved in enhancing cellulose degradation efficiency through the improved functional performance of cellulolytic bacteria. In the following sections, we elaborate on the specific mechanisms and representative cases of various synthetic biology strategies employed to advance cellulose degradation.

## Gene editing technologies facilitate cellulose degradation

**Fig. 2:**
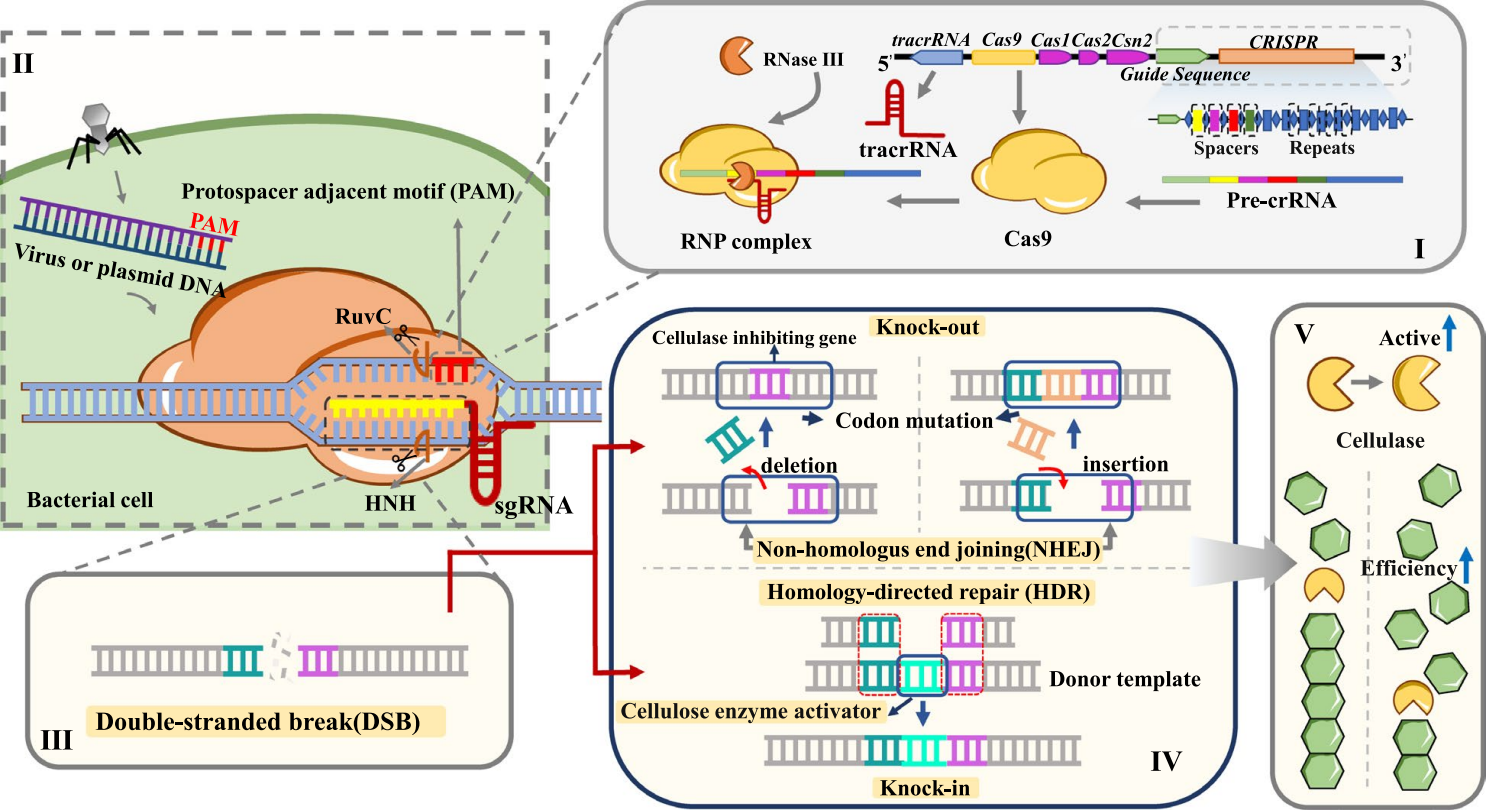
CRISPR-Cas9 mechanism for enhanced cellulase activity. CRISPR is a specific DNA sequence in the genome that contains fragments of foreign viruses or plasmids, which serve as a memory allowing bacteria to recognize and defend against the same invaders in the future. Cas9 is a protein that acts as the scissors in the CRISPR system (RuvC, HNH). When the CRISPR system detects the invading virus or plasmid DNA, Cas9 is guided to the target DNA sequence and then cleaves it, thereby destroying the exogenous DNA. This allows the DNA fragment to undergo non-homologous end joining repair or homologous recombination repair, achieving gene knock-in and knock-out, and resulting in the enhancement of cellulase enzyme activity. Abbreviations: CRISPR, Clustered Regularly Interspaced Short Palindromic Repeats; RuvC, RuvC protein; HNH, His-Asn-His nuclease domain; sgRNA, single guide RNA; crRNA, CRISPR RNA; tracrRNA, trans-activating CRISPR RNA; Cas, CRISPR-associated proteins; NHEJ, non-homologus end joining; HDR, homology-directed repair

Gene editing enables precise modification of nucleic acid sequences to alter genetic information and subsequent phenotypic traits. The key technologies encompass zinc finger nuclease technology (ZFN), transcription activator-like effector nuclease technology (TALEN) and clustered regularly interspaced short palindromic repeats-associated nuclease technology (CRISPR-Cas9) [104, 105]. In contrast to earlier technologies, CRISPR-Cas9 utilizes RNA–DNA base pairing for DNA recognition. This fundamental mechanism underpins its superior performance, which includes simpler design, higher efficiency and mutagenesis rates, reduced cytotoxicity and costs, as well as a greater capacity for multiplexed editing [106]. The mechanism of CRISPR-Cas9 gene editing technology involves the use of an artificially designed single-guide RNA (sgRNA) that identifies the target genomic sequence and directs the Cas9 endonuclease to cleave the DNA at the specified gene site, resulting in double-strand breaks. These breaks are subsequently repaired by cellular repair mechanisms, ultimately achieving the goal of modifying genomic DNA [107]. This technology exhibits considerable versatility in its applications, enabling diverse genetic manipulations such as gene knock-in, knock-out and multiplex genome editing (Fig. 2). Consequently, CRISPR-Cas9 holds significant promise for the genetic engineering of cellulose-degrading bacteria.

For instance, exo-β-1,4-glucanase acts as the rate-limiting enzyme in the cellulose degradation process, while the expression levels of endo-β-1,4-glucanase and β-glucosidase also significantly influence the overall efficiency of cellulose breakdown [76, 108]. CRISPR-Cas9 can be utilized to genetically modify key enzymes, augment the activity of restriction enzymes, knock out inhibitory genes in cellulose-degrading bacteria and supplement deficient cellulase gene expression, thereby enhancing the overall efficiency of cellulose degradation.

### CRISPR-Cas9-mediated modification of cellulose-degrading bacteria

**Fig. 3:**
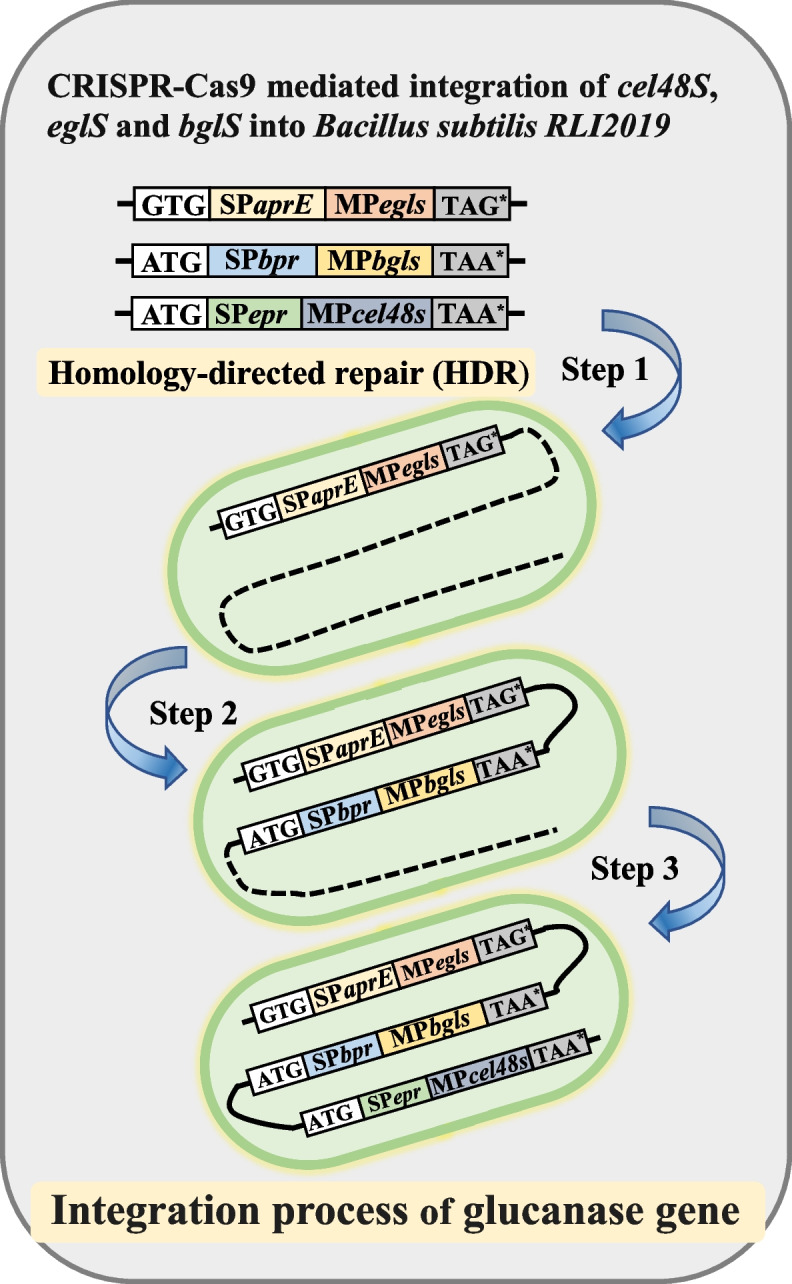
Homologous recombination repair of *Bacillus subtilis* by CRISPR-Cas9 technology. The genome of *Bacillus subtilis* RL2019 was engineered using CRISPR-Cas9-mediated editing to sequentially integrate the endogenous endoglucanase genes *eglS* and *bglS*, along with the exogenous *cel48S* gene from *Clostridium thermocellum*, into four specific loci: *aprE*, *bpr*, *nprE*, and *amyE*. This approach aimed to identify the most favorable integration site for optimal expression. Following the evaluation of cellulolytic activity, the most effective construct was selected and further employed for iterative insertion of the three target genes via self-homologous recombination repair, thereby achieving multi-copy integration within the *Bacillus subtilis* RL2019 genome. Abbreviations: *eglS*, endo-1,4-β-glucanase; *bglS,* β-glucosidase; *aprE*, serine alkaline protease (Ca^2^^+^-dependent subtilisin E); *bpr*, bacillopeptidase F; *nprE*, neutral metalloprotease NprE; *amyE,* α-amylase

The genus *Bacillus* found in the intestinal and rumen microbiota of livestock, serving as a probiotic that plays a crucial role, such as enhanced cellulose digestion, gut barrier reinforcement, oxidative stress reduction, microbiome modulation, and pathogen inhibition [109–112]. However, *Bacillus subtilis* shows deletions in key cellulose-degrading genes, particularly exo-glucanases, which impair its enzymatic efficiency and reduce its ecological adaptability in complex gut environments [113]. Moreover, its endo-glucanase expression was insufficient [48]. To address the functional limitations of both endogenous and exogenous glucanase genes, Liu et al. [114, 115] employed a CRISPR-Cas9-mediated iterative knock-in strategy to integrate and amplify multiple cellulolytic enzymes. This included inserting the exogenous exoglucanase gene *cel48S* from *Clostridium thermocellum* and increasing the copy numbers of the endogenous endoglucanase genes *eglS* and *bglS* into the most active expression sites (*aprE*, *bpr*, *nprE*, and *amyE* genomic loci) of *Bacillus subtilis*, resulting in the engineered strain *Bacillus subtilis* AEA3 (Fig. 3). The genetically engineered *Bacillus subtilis* strain AEA3 demonstrated substantially enhanced cellulolytic activity, with endoglucanase, exoglucanase, β-glucosidase, xylanase, and total cellulase levels increasing by 3.1-, 6.6-, 3.0-, 1.2-, and 1.8-fold, respectively. Given its high abundance in the animal intestine and rumen, coupled with strong colonization capacity, this strain holds strong promise as a direct-fed microbial (DFM) for enhancing fiber utilization in livestock receiving high-fiber diets. Nevertheless, critical challenges remain regarding the stable inheritance of engineered traits and the facilitation of synergistic interactions with other microbial communities, such as lignin- and hemicellulose-degrading bacteria, to maximize the utilization of complex fibrous substrates. These aspects represent essential directions for future research.

Research on CRISPR-Cas9 mediated editing of cellulase genes for animal husbandry remains at an early stage. Nevertheless, following CRISPR-Cas9 editing, *Bacillus subtilis* has been shown to utilize its biofilm more effectively to enhance cellulose degradation while concurrently inhibiting methanogenic activity and reducing methane production [110, 116]. Li et al. [117] utilized CRISPR-Cas9 to knock out the xylose transport inhibitor gene *araR* in *Bacillus licheniformis*, resulting in a significant increase in xylose degradation rates by the strain, which is of great significance for the degradation of hemicellulose-rich feed materials. Similarly, Xin et al. [118] applied CRISPR-Cas9 to disrupt the protease genes *aprE*, *nprE*, and *wprA*, along with the restriction endonuclease gene *bamHIR*, in *Bacillus amyloliquefaciens*, leading to increased α-amylase secretion and improved starch degradation efficiency. These successful genomic modifications in *Bacillus*-related strains demonstrate the potential of CRISPR-Cas9 technology to generate microbes that promote fiber degradation and mitigate methane emissions in agricultural applications.

### CRISPR-Cas9 mediated modification of cellulose-degrading fungi

Fungi represent a minor fraction of rumen microbiota, comprising approximately 10% [119]. To date, there have been no reported instances of CRISPR-Cas9 directly editing fungi within the gastrointestinal tract of livestock. Nevertheless, fungi serve as a critical source of exogenous enzymes that facilitate cellulose degradation in ruminants [120]. *Trichoderma reesei*, a filamentous fungus, is a representative species known for its robust ability to secrete cellulase for industrial biotechnological applications. Rangel Pedersen et al. [121] isolated and cultured *Trichoderma reesei* and performed monosaccharide analysis, confirming its capability to degrade various cell wall components found in different cotyledonous plant raw materials utilized as animal feed. Sakita et al. [122] observed that lambs fed with forage treated with *Trichoderma reesei* cellulase extract (FEE) showed significant increases in body weight and cellulose degradation rates, along with reduced methane (CH_4_) emissions. To enhance the efficiency and activity of cellulases produced by *Trichoderma reesei*, Zhang et al. [123] developed an efficient CRISPR-Cas9 system based on a tRNA-sgRNA multi-guide RNA platform tailored for this fungus. They designed two sgRNAs targeting *cre1* (encoding the carbon catabolite repressor 1 [124]) to knock-out *cre1*, while overexpressing the key cellulase/xylanase activator xyr1-A824V at the same genomic loci. This dual modification led to a notable increase in the activity of secreted cellulases. The digestion and absorption of cellulose have long presented challenges in feed science and animal nutrition. In conjunction with previous research findings [121, 122], CRISPR-Cas9 engineered *Trichoderma reesei* represents a promising source of exogenous cellulolytic enzymes. The highly active enzyme preparations produced by this engineered *Trichoderma reesei* can be directly used as feed additives to treat coarse feedstuffs. They are expected to play an increasingly important role in enhancing feed efficiency and maintaining the intestinal health of livestock.

CRISPR-Cas9 not only promotes adaptive evolution of cellulolytic fungi in specific environments [110], but also provides novel perspectives by integrating synergistic interactions observed in microbial ecology among enzymes and between enzymes and substrates [125]. Under this principle, it is revealed that CRISPR-Cas9 edited cellulose-degrading microorganisms can expose their effects on their own cellulase synthesis through substrates and inducers. The gene *Trctf1* (Trirerut 30:10530) was confirmed to exert a negative regulatory effect on cellulase synthesis [126]. Building on this, Chen et al. [126] used the CRISPR-Cas9 genome editing system to knock-out the transcriptional repressor *Trctf1* in *Trichoderma reesei* and induced the determination of cellulase activity with different inducers. The results showed that under lactose induction, the activities of cellulase, cellulose hydrolase, and endo-dextranase increased by 20.2%, 12.4%, and 12.9%, respectively. In contrast, no significant differences were observed when induced with a mixture of glucose and sophorose (MGD), and the activity of β-glucosidase showed no significant changes under different inducers. These cases clearly demonstrate that gene editing technology is transforming feed microorganisms from products of natural selection into intelligently designed products, tailored to specific needs, significantly expanding the technical arsenal for regulating animal nutrition.

## Development and utilization of cellulosomes

Cellulosomes are intricate, multi-enzyme complexes employed by anaerobic bacteria and fungi to efficiently hydrolyze cellulose in plant biomass. The gastrointestinal tract of livestock is predominantly colonized by anaerobic microbiota [127]. Accordingly, the assembly and function of cellulosomes are essential for microbial cellulose utilization and for sustaining intestinal health in these animals. Current strategies primarily include designer cellulosomes and artificial cellulosomes, both of which center on the rational engineering of scaffoldin proteins. By engineering scaffoldins to integrate multiple high-affinity cellulases, the catalytic efficiency and structural stability of cellulosomes can be significantly enhanced.

### Designer cellulosomes

**Fig. 4:**
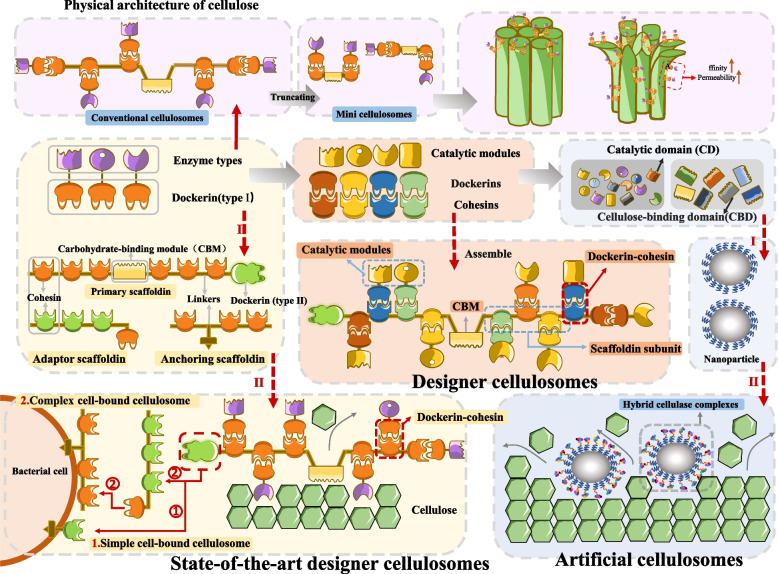
Architecture and engineering of natural and synthetic cellulosomes. Cellulosome consists of two main components: the catalytic and non-catalytic subunits. The non-catalytic subunit serves as a structural scaffold containing multiple cohesin modules, whose mechanical stability directly modulates cellulolytic activity. The catalytic subunit consists primarily of glycoside hydrolases connected to CBMs and dockerin domains, which collectively enable substrate targeting and enzyme integration into the complex. Through dockerin-cohesin interactions, the enzyme subunits are firmly anchored to the scaffold protein. Dockerin II mediates cell attachment, and the CBM recognizes cellulose. Subsequently, the catalytic subunits on the scaffold degrade the cellulose. Minicellulosomes can achieve high substrate binding affinity and enhanced permeability Designer cellulosome, formed by combining a scaffold protein composed of a chimeric protein scaffold made from adhesin modules derived from different bacterial species with various enzymes. Artificial cellulosome, magnetic nanoparticles (non-scaffold proteins) anchor to mini-scaffold proteins and bind to free enzymes. A modular library comprising CD of GH families and the binding domains (CBD) of the 30 CBM families. Abbreviations: CD, catalytic domains; GH, glycoside hydrolase; Other abbreviations as in Fig. 1

Currently, two principal strategies guide the development and application of designer cellulosomes. The first entails truncating natural scaffoldin proteins, as shown by Krauss et al. [128], to produce minicellulosomes that significantly enhance crystalline cellulose degradation efficiency. The second strategy adopts structural principles from natural cellulosomes [89, 90] (Fig. 4), employing chimeric scaffold proteins composed of cohesin modules sourced from diverse bacterial species integrated with multiple enzymes to assemble synthetic cellulosomes [129]. For example, Morais et al. [130] designed a chimeric scaffold incorporating three distinct cohesins from *Archaeoglobus fulgidus* (G), *Clostridium thermocellum* (T), and *Clostridium clariflavum* (V) (Fig. 4). This engineered scaffold exhibits exceptional thermal stability upon cellulase binding and demonstrates superior degradation performance compared to conventional cellulosomes, thereby improving adaptability to extreme conditions. Both approaches incorporate modifications originating from *Clostridium thermocellum*. This design enhances the stability of enzyme systems under the harsh conditions encountered during feed processing, such as elevated temperatures during pelleting, as well as within the complex gastrointestinal environment.

In addition to the aforementioned *Clostridium* species, other prevalent anaerobic bacteria in the gastrointestinal tract that produce cellulosomes include *Ruminococcus* [131], *Acetivibrio* [132], *Bacteroides* and *Pseudobacteroides* [133]. The cellulosome of *Ruminococcus flavefaciens* exhibits particularly high complexity. Recently, Duarte et al. [134] employed X-ray crystallography to reveal that a single cohesin domain from *Ruminococcus flavefaciens* can bind two short dockerin domains simultaneously. Their work also elucidated the structural and functional mechanisms of truncated dockerins. Furthermore, truncated dockerin variants have been engineered successfully in other bacteria, including *Bacteroides cellulosolvens* and *Clostridium thermocellum*. For synthetic cellulosome design, adopting short dockerins may lower production costs by allowing a single scaffold protein to recruit multiple enzymes, thereby increasing enzymatic density. Building on the proven utility and design flexibility of this approach in *Clostridium thermocellum* [135], we propose the design of compact multi-enzyme complexes by integrating multiple CBMs and catalytic domains. Such architectures are expected to concurrently achieve high substrate binding affinity and enhanced permeability, thereby improving their capacity to modify the physical structure of cellulose. This approach presents a novel strategy for developing cost-effective feed enzyme preparations (Fig. 4).

### Artificial cellulosomes

Inspired by the self-assembly and multi-enzyme synergy of natural cellulosomes [90], artificial cellulosomes have been developed as biomimetic supramolecular complexes. These systems rely on specific intermolecular interactions among biomolecules for their formation. Artificial cellulosomes can be categorized into protein-based and non-protein-based systems based on the scaffold type. The following section will focus specifically on those employing non-protein scaffolds.

The mutant strain SM901 of *Clostridium thermocellum* lacks the scaffold protein CipA, which prevents the assembly of natural cellulosomes. To address this, Krauss et al. [128] successfully assembled artificial cellulosomes by immobilizing mini-scaffoldins onto magnetic nanoparticles and subsequently recruiting free enzymes. In 2024, a team led by Hikaru Nakazawa [136] developed an artificial hybrid cellulase complex using a modular library approach. They constructed a library comprising 49 catalytic domains (CDs) from glycoside hydrolase (GH) families and 30 carbohydrate-binding domains (CBDs) from CBM families (Fig. 4). From this library, nine highly active CDs and 24 CBDs were selected for nanoparticle assembly. Synergistic interactions among CBDs considerably enhanced cellulolytic activity. This study provides the first systematic evidence that modular combinatorial screening can exceed the performance of natural enzyme systems, offering a novel design strategy for highly efficient multi-enzyme complexes. This plug-and-play strategy facilitates the development of specialized enzyme formulations tailored to specific feed ingredients, such as corn cobs and soybean meal. In the future, these engineered complexes may be directly incorporated into animal feed or coated for rumen regulation in ruminants, thereby enabling synergistic degradation of cellulose.

Currently, the research and application of artificial cellulosomes in the field of animal husbandry remain limited. Building upon Krauss’s work on the modification of *Clostridium thermocellum* strains lacking scaffold proteins [128], future work may focus on overcoming the inability of certain intestinal anaerobic bacteria to naturally assemble cellulosomes, thereby enabling the construction of artificial cellulosomes. These artificial structures could be combined with modularly designed cellulase complexes and incorporated into enzyme preparations or feed ingredients to promote synergistic cellulose degradation.

### Application of AI technology in the field of cellulose degradation

With artificial intelligence (AI)'s expanding role across diverse disciplines [137, 138], its integration into synthetic biology has become increasingly essential. Within cellulose degradation research, a major challenge lies in the efficient and rapid identification of optimal microbial strains during the isolation, screening, and cultivation of cellulose-degrading microorganisms. This bottleneck often impedes subsequent microbial engineering via synthetic biological approaches. The implementation of AI technologies offers a promising avenue to address this issue by facilitating the rapid selection and culture of high-performance strains.

In 2024, Luong and Poeaim [139] employed an artificial neural network-genetic algorithm (ANN-GA) approach to characterize the novel lignocellulose-degrading fungus *Pseudolagarobasidium acaciicola* TDW-48, recently identified in Thailand. They established a nonlinear mapping model linking fermentation parameters to the activities of multiple enzymes, filter paper enzyme, endoglucanase, β-glucosidase, and xylanase. Utilizing the ANN model as a fitness function, the study optimized enzyme activities, notably achieving a significant enhancement in β-glucosidase activity. This approach effectively mitigated the inhibitory effects of cellobiose on both endoglucanase and exoglucanase activities. Recently, Mao et al. [140] applied graph neural networks (GNNs) to reconstruct regulatory pathways linking quorum sensing (QS) signals to degradation genes within microbial communities. By integrating synthetic biology techniques with these computational models, their work holds promise for elucidating the mechanisms and regulatory networks of cellulose-degrading bacteria, such as the degradation of crystalline fibers by *Fibrobacter succinogenes*, which remain largely unexplored. Concurrently, Wang et al. [141] introduced EasIFA, an enzyme active site annotation algorithm with substantial potential for catalytic site monitoring. This tool facilitates the design of enzymes with functionalities surpassing. AI technology, as an efficient tool, has effectively solved the difficulties in screening and isolating cellulose microorganisms. By using AI algorithms to screen and predict target strains, it has laid a solid foundation for subsequent applications of synthetic biology technology to modify the target strains.

## Prospect

Efficient cellulose degradation represents a central challenge for enhancing animal husbandry productivity and achieving sustainability. Current findings demonstrate that synthetic biotechnologies hold considerable potential in cellulose degradation, offering a promising breakthrough, which enables the targeted construction of microbial strains and precise regulation of enzymatic and microbial activity, drawing on strategies such as cellulase gene editing, designer multi-enzyme complexes, and AI-driven algorithms. Future efforts should focus on optimizing a broader range of enzymatic parameters and expanding application scenarios through molecular-level engineering.

CRISPR-Cas9 technology is characterized by high efficiency, precision, low cost, and broad adaptability, substantially improving cellulose degradation efficiency. It enables not only microbial genome engineering but also extends to applications such as disease modeling and drug discovery. However, CRISPR-Cas9 is associated with off-target effects that can lead to unintended cleavage and disruption of non-target genes [142]. Safe and efficient delivery of editing tools into target cells remains challenging. Recently, Liu et al. [143] achieved highly efficient multiplex gene editing in *Bacillus licheniformis* using the CRISPR-Cpf1 system, demonstrating high knockout success rates. This novel CRISPR-Cpf1 system holds promise for enabling more precise and reliable genetic engineering in cellulose-degrading bacteria.

Designer and artificial cellulosomes represent key experimental systems for studying cellulosome structure and function. Chimeric scaffoldins, engineered from cohesin modules of diverse bacterial origins, can markedly enhance catalytic performance and stability under extreme conditions. These assemblies are not only relevant to the livestock sector but are also employed in consolidated bioprocessing (CBP) for biofuel production. Due to the complexity of cellulosomal enzyme systems, major challenges persist, including functional characterization of numerous protein components and the high cost of non-protein scaffolds. AI offers promising approaches to address nonlinear optimization challenges in such complex biological processes. While the potential of these technologies is considerable, their limitations must also be acknowledged. The economic feasibility and scalability of AI-assisted solutions remain to be thoroughly evaluated.

In prospective applications, these technologies can be converged and synergized to establish a closed-loop technological framework that capitalizes on their respective advantages while addressing existing limitations. Artificial intelligence can be utilized to predict and screen for highly efficient cellulose-degrading microorganisms. Through gene editing and cellulosome engineering, genetically enhanced microbes or enzyme complexes may be introduced into feed or administered as probiotics or enzyme supplements within the intestinal tract. After digestion, AI can again assist in screening for functional cellulose-degrading microorganisms from the livestock digestive tract and feces. This cyclic strategy aims to achieve efficient cellulose degradation, promote intestinal health, improve feed digestibility, reduce operational costs, and ultimately foster a sustainable circular bioeconomy.

While conventional synthetic biology approaches have predominantly focused on editing individual isolates, natural environments (such as soils, animal gastrointestinal tracts and manure) are highly intricate ecosystems where single engineered strains often face challenges in survival and functionality. Under these conditions, a top-down strategy employing synthetic microbial communities (SynComs) has emerged as a promising alternative. SynComs provide an ideal platform for facilitating synergistic interactions among diverse microorganisms [144]. Through the rational selection of environmental variables (e.g., enrichment cultures, artificial selection or directed evolution) the resilience and functional capacity of microbial strains can be enhanced [145]. This approach alleviates the metabolic burden on individual members while collectively improving degradation efficiency. As an extension of synthetic biology principles to the ecological level, SynComs represent the application of engineered microbial consortia for community-level functions. The continued integration of such strategies will advance the field from designing single cells toward an era of programming entire ecosystems.

Furthermore, synthetic biology technologies hold significant potential for targeting the degradation or utilization of other non-conventional carbohydrates in numerous microbial-host health modulation pathways. For instance, precise adjustments of the degradation preferences of gut microbiota through gene editing and microbial resynthesis can yield multiple benefits beyond energy acquisition. Yu et al. [146] demonstrated that *Bacteroides thetaiotaomicron* and *Bacteroides ovatus* exhibit distinct fructan utilization preferences for levan and inulin, respectively, and that these bacteria further induce divergent dietary preferences in their hosts. Using gene editing, they replaced the fructan utilization genes in *Bacteroides thetaiotaomicron* with those from *Bacteroides ovatus*, reprogramming its substrate preference from levan to inulin and thereby reversing the dietary preferences of colonized mice. These findings reveal that gut bacteria modulate host intake of specific complex carbohydrates through their specialized metabolic traits. This regulation is not merely dependent on bacterial capability, but is an integrated process involving host energy sensing and neural responses mediated by the hypothalamic arcuate nucleus, rather than relying exclusively on classical short-chain fatty acid production. Collectively, synthetic biology approaches are providing novel insights into how gut microbial metabolic activities shape host energy perception and dietary behavior.

## Summary

Synthetic biology is transforming our understanding and utilization of microorganisms for cellulose degradation. It transcends mere imitation of natural processes, representing instead a significant redesign and recreation of living systems. Techniques such as CRISPR-Cas9 enable targeted gene editing, knocking in beneficial genes (e.g., endoglucanases, β-glucanases, and exogenous cellulases) and knocking out limiting factors (e.g., *Trctf1*), to boost microbial performance and cellulase expression. Cellulosome efficiency has been advanced through engineered scaffoldins, microbial adhesion modules, nanoparticle integration, and optimized dockerin domains. Furthermore, synthetic biology facilitates the prediction of fermentation parameters, elucidation of enzymatic mechanisms, and design of regulatory circuits. In the future, we can anticipate several key developments: Tailored probiotics and enzyme preparations are expected to become standard components of precision nutrition programs. Synthetic microbial communities will likely be widely adopted as living biological drugs and nutritional supplements for the management of animal health and AI based real-time nutritional regulation models will dynamically adjust feed formulations based on the gut microbiota status of individual animals. Acting as a powerful convergent force in technology, synthetic biology is poised to serve as the core engine driving advancements in animal science and agricultural biotechnology into a new era, thereby making significant contributions toward achieving sustainable agricultural development goals.

## Data Availability

No datasets were generated or analysed during the current study.

## Abbreviations

BG: β-Glucosidase
Cas: CRISPR-associated proteins
CBH: Exo-β-1,4-glucanase
CBM: Carbohydrate-binding module
CCR: Carbon catabolite repression
CD: Catalytic domains
CRISPR: Clustered Regularly Interspaced Short Palindromic Repeats
crRNA: CRISPR RNA
DFM: Direct-fed microbial
EG: Endo-β-1,4-glucanase
GH: Glycoside hydrolase
HDR: Homology-directed repair
HNH: His-Asn-His nuclease domain
NHEJ: Non-homologus end joining
NR: Non-reducing end
R: Reducing end
RuvC: RuvC protein
sgRNA: Single guide RNA
tracrRNA: Trans-activating CRISPR RNA
